# The application of electroencephalogram in depression research: bibliometric and technological application analysis from 2005 to 2025

**DOI:** 10.3389/fnins.2025.1653693

**Published:** 2025-08-21

**Authors:** Yican Hao, Yanli Han, Jian Huang, Cangcang Hao, Bo Yu, Shenting Wei, Kuiyan Zhou

**Affiliations:** ^1^Department of First Clinical Medical College, Shandong University of Traditional Chinese Medicine, Jinan, China; ^2^The Second Affiliated Hospital of Shandong University of Traditional Chinese Medicine, Jinan, China; ^3^Handan Maternal and Child Health Hospital, Handan, China

**Keywords:** electroencephalogram, depression, diagnosis, bibliometrics, visual analysis

## Abstract

**Background:**

Depression is a common mental disorder, and its diagnosis is highly dependent on subjective assessment. Electroencephalogram (EEG), as a non-invasive and economical neurophysiological tool, has garnered considerable attention in recent years in the research of auxiliary diagnosis and clinical application. However, there exists a limited number of articles that summarize this body of research. This study aims to investigate the current trends, emerging topics, and potential advancements in EEG research related to depression while also predicting the challenges that may arise within this field.

**Methods:**

We retrieved the literature related to depression and EEG published from April 16, 2005 to April 16, 2025 in Web of Science (WoSCC) and PubMed, and conducted data analysis and visual display using CiteSpace, VOS viewer, Bibliometrix, Scimago Graphica, Microsoft Excel 2021, and R software version 4.2.3.

**Results:**

From 2005 to 2025, 215 journals from 189 countries published papers in this field. The majority of the papers were published in *Journal of Affective Disorders*, and the average citation per paper was the highest in *Biomedical Signal Processing and Control*. China contributed the most publications, but the United States had the highest citation per paper. In terms of the total number of publications, Lanzhou University contributed the most papers. The top 5 keywords were major depression, alpha asymmetry, brain, asymmetry, and anxiety. Cluster analysis indicated that the research in this field is transforming from basic electrophysiological features to clinical applications, that is, exploring the significance of EEG in the diagnosis, classification, and prediction of depression.

**Conclusion:**

EEG research on depression is developing toward individualization and intelligence. In the future, efforts should be focused on standardizing processes, integrating multiple modalities, and clinical application to enhance its value in diagnosis and prognosis.

## 1 Introduction

Depression is a highly prevalent mental illness worldwide and is considered one of the leading contributors to global disability-adjusted life years (DALYs) and years lived with disability (YLDs) (Ferrari et al., 2024). In 2008, the World Health Organization (WHO) identified depression as the third largest contributor to global economic burden and projected that it would become the leading cause by 2030 (Malhi and Mann, 2018; Mathers and Loncar, 2006). The core symptoms of depression include persistent low mood, cognitive impairment, and increased risk of suicide, all of which severely affect patients’ quality of life and social functioning. Currently, the diagnosis of depression predominantly depends on clinical symptomatology and psychological assessment scales, as there are no universally accepted objective or quantifiable biological markers. As the number of patients with depression continues to grow and the clinical presentations become increasingly diverse, traditional diagnostic approaches based on subjective evaluation face significant risks of misdiagnosis and underdiagnosis, highlighting the urgent need to identify reliable biomarkers (Zhou et al., 2021).

Electroencephalogram (EEG), as a non-invasive, cost-effective, and easy-to-operate neurophysiological tool, captures real-time patterns of brain activity and has been widely used in neuroscience research (Babiloni et al., 2021; Hirschauer et al., 2015). In recent years, with advancements in EEG signal processing, the growing accessibility of portable acquisition devices (e.g., three-lead EEG), and the rapid development of artificial intelligence-assisted diagnostic tools (such as machine learning methods), EEG has attracted increasing attention for its potential role in the early screening and auxiliary diagnosis of depression (Fingelkurts and Fingelkurts, 2015). This technique enables multidimensional analysis of various neurophysiological signals, including resting-state EEG, task-related EEG, and event-related potentials (ERPs). Resting-state EEG reflects the brain’s spontaneous activity during a quiet wakeful state and is rich in information about intrinsic neural networks (e.g., the default mode network), which is useful in identifying functional connectivity abnormalities associated with depression. Task-related EEG captures neural dynamics while individuals engage in specific cognitive or emotional tasks, aiding in the detection of deficits in emotion regulation and cognitive processing. ERPs, which measure the averaged brain response to specific external stimuli, offer insights into higher-order cognitive functions such as attention, memory, and emotion recognition, and are frequently used to explore information processing in patients with depression. Compared with functional magnetic resonance imaging (fMRI)—a widely applied technique in this field—EEG offers superior temporal resolution, making it uniquely suited to detect transient brain activity associated with emotional fluctuations. As such, EEG has become a key tool in interdisciplinary research on neuropsychiatric disorders (Fleury et al., 2023).

This study employs bibliometric analysis to systematically examine the research output on EEG applications in the field of depression from 2005 to 2025, aiming to uncover trends in technological development, emerging research hotspots, and the potential value of EEG in clinical and basic research. The findings are intended to provide theoretical grounding and data support for future studies and clinical applications.

## 2 Materials and methods

### 2.1 Data sources and search strategies

**Figure 1:**
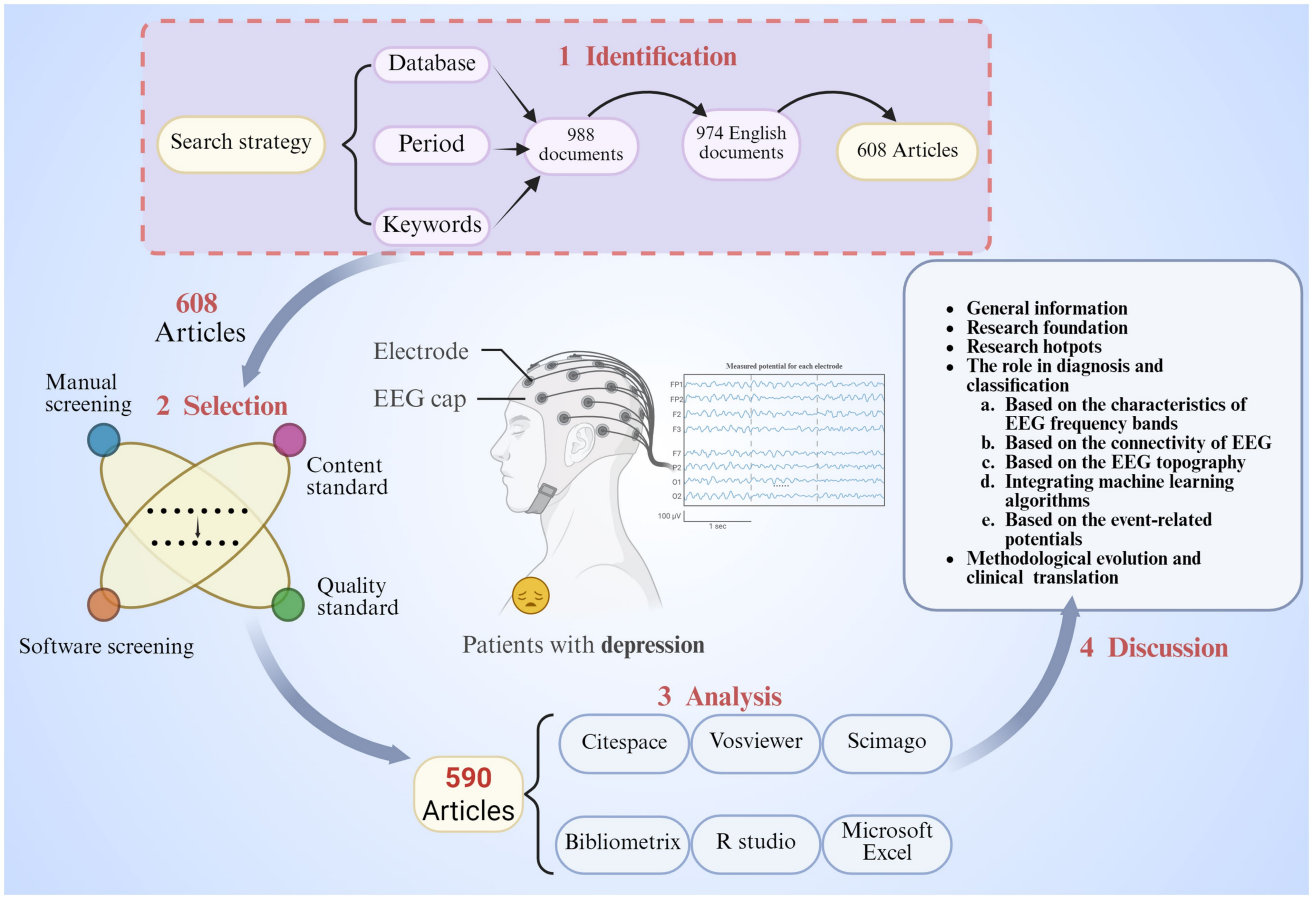
The detailed flowchart of the literature (By BioRender, publication license number: XV28MG2BR2).

In this study, data related to EEG and depression were retrieved from the Web of Science Core Collection (WoSCC), a highly esteemed and influential citation database that facilitates bibliometric analysis. The search was conducted on April 16, 2025, using the following search strategy: TI = [(“EEG” OR “Electroencephalogram*” OR “Electroencephalography”) AND (“depress*” OR “dysthymi*”)]. The search period was limited to publications between April 16, 2005, and April 16, 2025. A total of 988 records were initially identified, of which 974 were in English. We focused exclusively on publications classified as “articles,” resulting in the inclusion of 608 records. The detailed screening process is illustrated in Figure 1. In addition, we conducted necessary verifications on the research results. Using the same search strategy on PubMed, we obtained a total of 53 clinical trials related to electroencephalogram and depression.

### 2.2 Data extraction, cleaning and standardization

Data extraction, cleaning, and standardization were conducted independently by two researchers, with cross-validation conducted to ensure consistency. The literature search was completed on April 16, 2025, in a single day to minimize bias caused by database updates. To enhance the quality and thematic relevance of the publications included, an extensive manual screening was performed by the research team. This process was time-consuming and involved independent review of titles and abstracts by both researchers. Full texts were examined when necessary to assess eligibility. Studies not directly related to EEG or depression were excluded. Any discrepancies during the screening process were resolved through discussion and consensus with a third researcher. As summarized in Table 1, manual screening criteria were categorized into two dimensions: content standard and quality standard. The former ensured that articles were closely related to the research topic, while the latter confirmed adherence to basic academic standards in structure and formatting.

**Table 1:** Criteria for inclusion in the manual screening.

Type	Inclusion criteria
Content criteria	The included literature should encompass research topics related to depression
	Topics related to EEG, including the role of EEG in diagnostic markers, efficacy monitoring and intervention
Quality criteria	The literature should be at least two pages long
	The necessary components of an academic paper, such as the abstract, author details, keywords and references, must be fully included in the submitted documents
	Literature that does not incorporate the aforementioned elements will be excluded from consideration
	Double-blind peer review

All bibliometric data were derived from the WoSCC, with the search results including “complete records and cited references” and exported in “Txt” format. To minimize the risk of omission, the preliminarily screened records were imported into CiteSpace for systematic duplicate detection, which confirmed no redundant entries. To ensure accuracy and consistency in subsequent analysis, terminological variations (e.g., singular/plural forms, synonyms) were standardized, and duplicated terms were merged. Since the data format was compatible, no conversion was required prior to bibliometric analysis. The entire literature selection process strictly followed the PRISMA guidelines (Page et al., 2021). Ultimately, 590 studies meeting the inclusion criteria listed in Table 1 were included. A wide range of established bibliometric studies were referenced to ensure the robustness and statistical relevance of the selected dataset.

### 2.3 Bibliometric analysis

To systematically present and analyze the characteristics and trends of the included literature, multiple bibliometric tools were employed, including CiteSpace 6.4.R1, VOSviewer 1.6.19, Scimago Graphica 1.0.39, Microsoft Excel 2021, and an online bibliometric analysis platform. These tools facilitated a comprehensive depiction of the current state of research, highlighted key areas of focus, and revealed the developmental trajectory and evolutionary trends of EEG in depression studies. A graphical abstract was created using BioRender.^1^ National collaboration frequency and core journal distribution were calculated using the Bibliometrix software, with relevant data available at https://bibliometric.com. The national cooperation chord diagram was created using the Charticulator platform.^2^ VOSviewer^3^ was utilized to create and visualize scientific knowledge networks (van Eck and Waltman, 2010), including collaborations among institutions and authors, as well as publication volumes. In these networks, “Total Link Strength” indicates the total strength of connections between nodes, with higher values representing stronger collaborative relationships. CiteSpace^4^ was applied to identify research frontiers, thematic shifts, and emerging trends in the EEG field (Farahat and Elsaid, 2022). It enabled analysis of keyword hotspots, clustering, burst detection, citation patterns, and knowledge flow. Node betweenness centrality, a measure of a node’s bridging role within the network, was used to indicate influence. Nodes with higher centrality values are shown as larger circles with purple rims. Scimago Graphica Scimago Graphica was used to visualize country-level publication counts, citation frequencies, and relevance scores. Journal impact factors (IF) were obtained from the 2023 edition of the Journal Citation Reports (JCR) within the WoSCC database. Statistical analysis were conducted using R software (version 4.2.3, http://www.R-project.org) to explore economic factors associated with EEG-related depression research output, specifically gross domestic product (GDP) and GDP per capita. Economic data were sourced from the World Bank.^5^ The Shapiro–Wilk test was used to assess variable distribution, followed by analysis using Spearman’s rank correlation coefficient. A *p*-value <0.05 was considered statistically significant.

## 3 Results

### 3.1 Trends in publications

**Figure 2:**
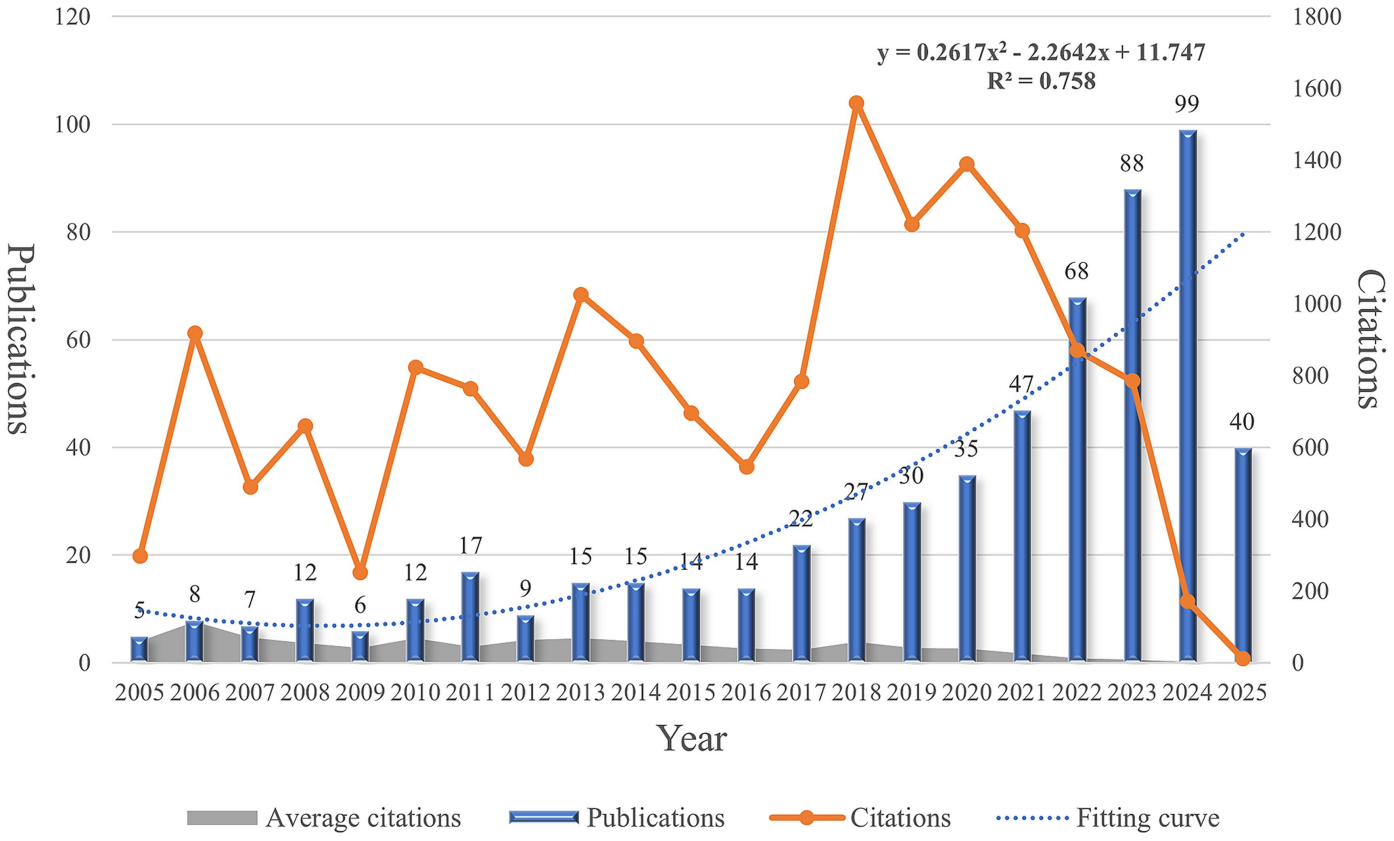
Annual publication and citation trends of articles on the use of electroencephalography in depression from 2005 to 2025.

Figure 2 presents the trends in annual publication output and citation frequency. Notably, the number of publications peaked in 2024, with a total of 99 articles. From 2005 to 2016, the annual publication volume remained relatively low, until 2017 when it first exceeded 20 articles. Although the publication count in 2018 was not the highest, that year recorded the highest number of total citations, indicating the significant impact of studies published during that period.

Since 2020, there has been a steady increase in annual publications; however, citation counts have shown a marked decline. Interestingly, publications from 2006 had the highest average number of citations per article, suggesting foundational contributions to the field. A second-order polynomial regression analysis revealed a statistically significant correlation between publication year and number of publications (*R*^2^ = 0.758), reflecting growing academic interest and research investment in the application of EEG in depression studies over the past two decades.

### 3.2 Analysis of countries/regions and institution

**Figure 3:**
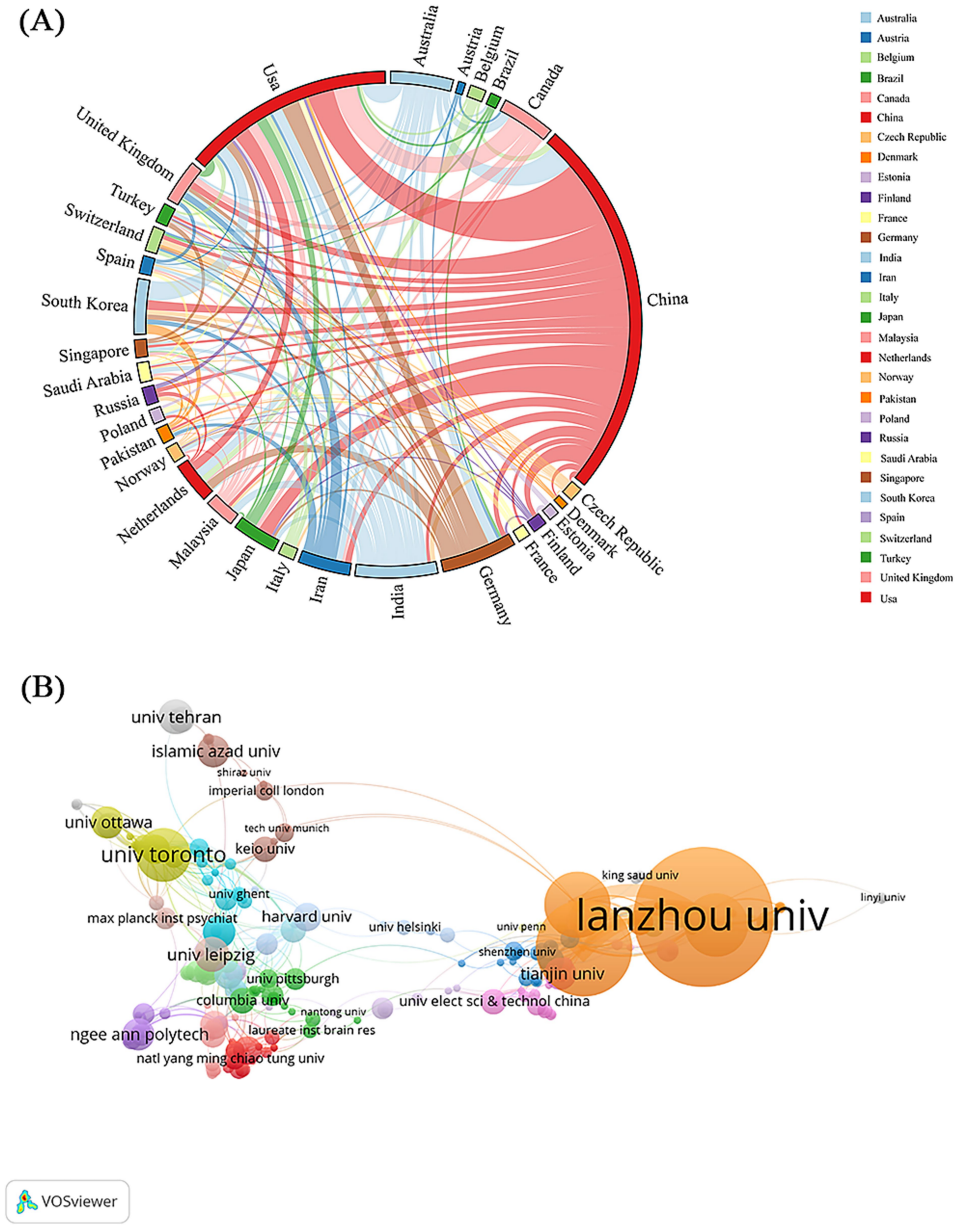
**(A)** Network diagram of cooperation among countries/regions. **(B)** Visualization of institutional cooperation.

A total of 2,066 institutions from 189 countries/regions have contributed to EEG research in the context of depression. As illustrated in Figure 3A, international collaboration is active, with China (214 publications), the United States (118), and India (46) emerging as the leading contributors. Major collaborations were observed between the U.S. and European countries (Figure 3A; Supplementary Figure S1). Supplementary Table S1 ranks the top 10 countries and institutions based on publication volume. Among the 189 countries, only two produced more than 100 publications: China, leading with 214 papers—nearly twice as many as the U.S. Despite China’s dominance in publication volume, the top five countries by total citations differ. The U.S. ranks first with 5,201 citations, followed by China (3,761), India (1,728), Germany (1,310), and Australia (1,139). The U.S. also ranked first in citation impact, with an average of 44.08 citations per article, demonstrating both high output and significant academic influence.

According to Supplementary Table S1, the top 10 most productive institutions produced a total of 177 publications, representing for 29.11% of all included studies. Lanzhou University ranked first, with 45 publications and 1,509 citations, demonstrating both high productivity and substantial academic impact. Figure 3B displays the co-authorship network among institutions. The size of each node indicates publication output, and the thickness of links reflects the strength of collaborations. Ten distinct clusters were identified, with Lanzhou University showing strong collaborative ties. Foundational contributions were also noted from Harvard University and the University of Helsinki (Supplementary Figure S2).

At the national level, a notable positive correlation was observed between the number of publications related to EEG and depression and a country’s GDP (Spearman rho = 0.725, *p* = 3.77 × 10^−10^). Similarly, per capita GDP also showed a statistically significant correlation with publication volume (rho = 0.277, *p* = 0.04079). These findings suggest that a country’s overall and per capita economic capacity may affect the volume and intensity of its research output in this field.

### 3.3 Analysis of authors and co-cited authors

**Figure 4:**
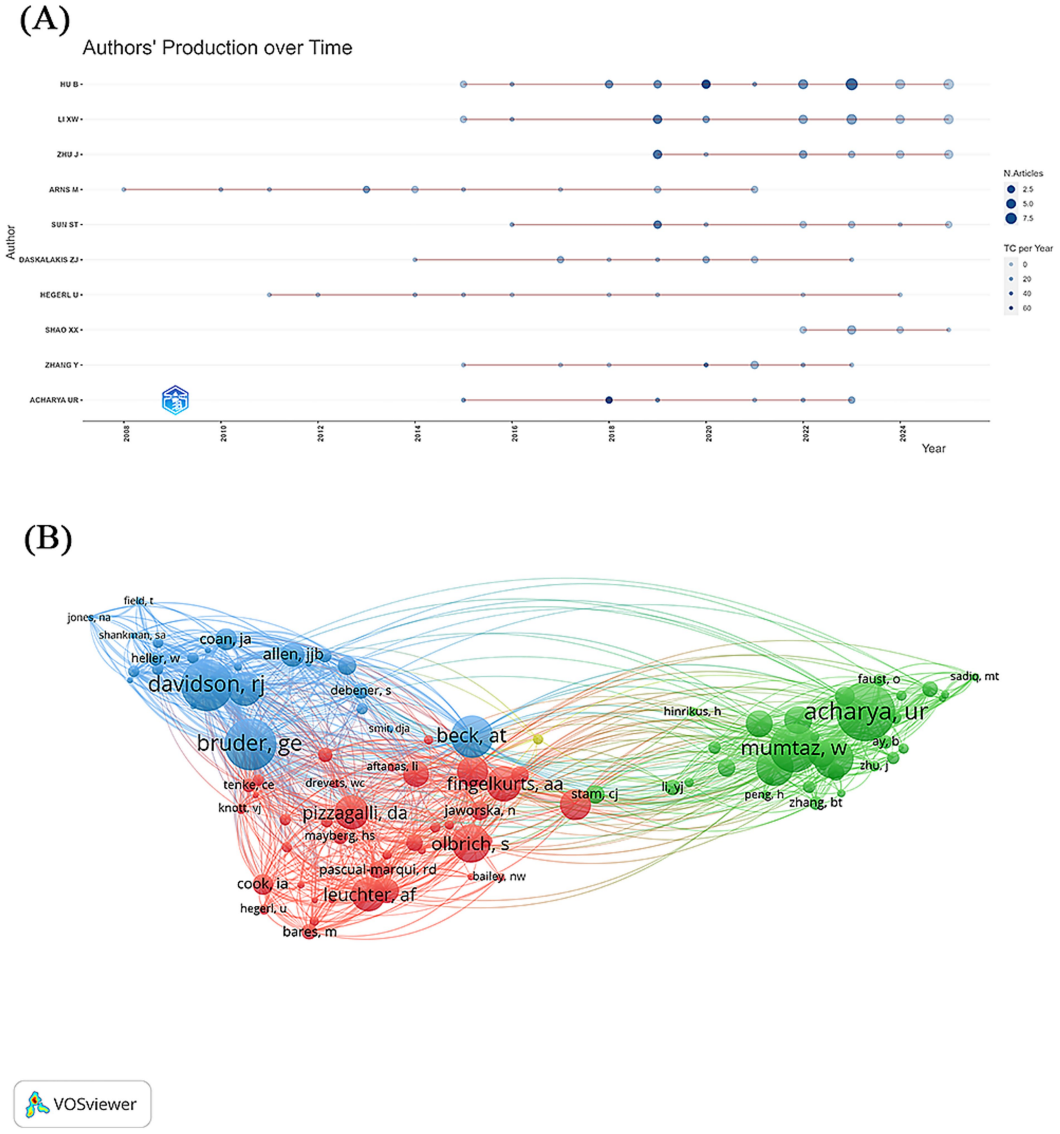
**(A)** Timeline chart of the top 10 most productive authors. **(B)** Visualization chart of the top 100 most cited authors.

Bibliometric analysis identified a total of 2,581 authors and 3,682 co-cited authors who contributed to the development of EEG research in depression. Table 2 and Figure 4A present the top 10 most prolific authors in this domain. Among them, Hu, B. emerged as the most productive author with 39 publications over the past two decades, followed by Li, X. W. (28 publications) and Zhu, J. (17 publications). Average citation count serves as an indirect indicator of the academic recognition of an author’s research. Among the top 10 authors, eight exhibited an average citation count exceeding 10. Hu, B. had the highest total citations (*n* = 254), whereas Acharya, U. R. had the highest average citations per article (14.38), highlighting the broad academic recognition of his research quality. The scientific influence of authors was further evaluated using the H-index, a metric that integrates both productivity and citation impact, offering a more comprehensive assessment of academic contribution (Roldan-Valadez et al., 2019). All top 10 authors demonstrated high H-index values, underscoring their central role in EEG-related depression research. In terms of co-citation analysis, Acharya, U. R. ranked first with 222 co-citations, identifying him as one of the most influential scholars in the field. Figure 4B illustrates the collaboration network among the top 100 co-authors, revealing close cooperative relationships among prolific researchers. These connections emphasize the pivotal role of core author clusters in advancing this area of research.

**Table 2:** The top 10 authors and co-citation authors in the field of EEG research related to depression.

Rank	Author	Documents	Citations	Average citations	H-index	Co-cited author	Citations
1	Hu, B.	39	254	6.51	16	Acharya, U. R.	222
2	Li, X. W.	28	238	8.5	12	Davidson, R. J.	196
3	Zhu, J.	17	186	10.94	11	Bruder, G. E.	195
4	Arns, M.	13	160	12.31	9	Mumtaz, W.	183
5	Sun, S. T.	12	158	13.17	9	Beck, A. T.	158
6	Daskalakis, Z. J.	10	133	13.3	7	Cai, H. S.	153
7	Hegerl, U.	9	128	14.22	7	Olbrich, S.	143
8	Shao, X. X.	9	126	14	7	Li, X. W.	139
9	Zhang, Y.	9	117	13	7	Fingelkurts, A. A.	134
10	Acharya, U. R.	8	115	14.38	7	Leuchter, A. F.	128

### 3.4 Visual analysis of journals and co-cited journals

Between 2005 and 2025, a total of 215 journals published articles pertaining to EEG and depression. To identify the core sources of publication in this field, Bradford’s Law was applied (Venable et al., 2016) As shown in Supplementary Figure S3, Bradford analysis via the Bibliometrix platform identified 11 core journals that collectively published 195 articles—accounting for 32.07% of all included studies. Table 3 lists the top 10 journals in this field based on the number of publications, along with their 2024 Journal IF from the Web of Science JCR. IFs are widely used to measure a journal’s academic influence and visibility (Roldan-Valadez et al., 2019). Three journals had IFs greater than 4; six were ranked in JCR Q1, three in Q2, and one in Q3. The *Journal of Affective Disorders* published the highest number of articles (*n* = 44) and received the most citations (*n* = 932). However, the highest average citations per article were observed for *Biomedical Signal Processing and Control* (*n* = 50.89), which ranked second in publication volume, reflecting its strong research influence. Among the top 10 co-cited journals, nine were classified as JCR Q1. The *American Journal of Psychiatry* stood out with an IF exceeding 15. Other highly cited journals—each receiving over 500 citations—included *Biological Psychiatry*, *Journal of Affective Disorders*, *NeuroImage*, *Clinical Neurophysiology*, and *Psychophysiology*, all serving as foundational sources of knowledge in this field.

**Table 3:** The top 10 journals and co-citation journals in the field of EEG research related to depression.

Rank	Sources	Articles	Citations	Average citations	JCR	Impact factor (2024)	Co-cited journal	Citations	JCR
1	Journal of Affective Disorders	44	932	21.18	1	4.9	Biological Psychiatry	932	1
2	Biomedical Signal Processing and Control	18	916	50.89	1	4.9	Journal of Affective Disorders	916	1
3	Clinical Neurophysiology	18	834	46.33	1	3.6	NeuroImage	834	1
4	Frontiers in Psychiatry	18	743	41.28	2	3.2	Clinical Neurophysiology	743	1
5	Biological Psychology	16	503	31.44	1	2.8	Psychophysiology	503	1
6	IEEE Access	16	405	25.31	2	3.4	Biological Psychology	387	1
7	Scientific Reports	16	387	24.19	1	3.9	Computer Methods and Programs in Biomedicine	365	1
8	Clinical EEG and Neuroscience	15	365	24.33	3	1.7	American Journal of Psychiatry	364	1
9	Frontiers in Neuroscience	13	364	28	2	3.2	International Journal of Psychophysiology	360	2
10	IEEE Transactions on Neural Systems and Rehabilitation Engineering	11	360	32.73	1	5.2	Human Brain Mapping	345	1

**Figure 5:**
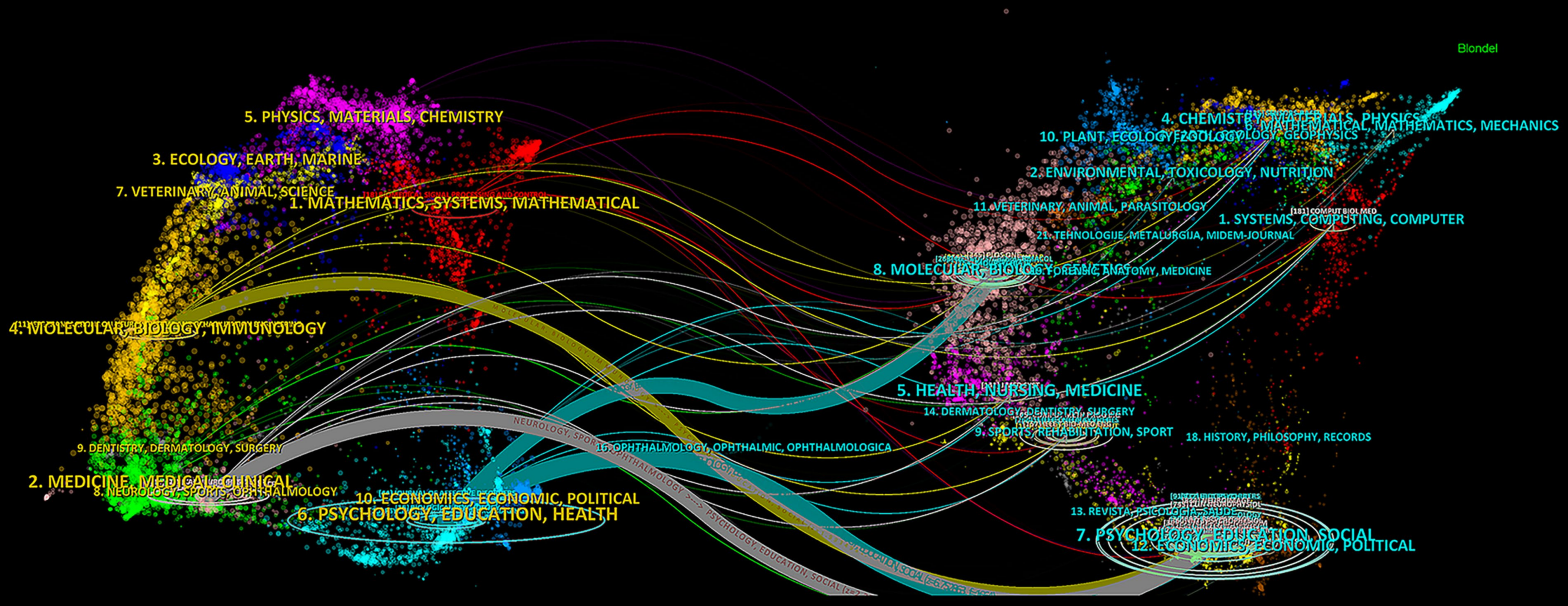
Visualization of dual-map overlay for journals.

In addition, a journal dual-map overlay analysis was employed to explore the evolution of disciplinary structures and identify emerging research frontiers (Chen and Leydesdorff, 2014). Figure 5 visualizes citation pathways between citing and cited journals from 2005 to 2025. The left side represents the cited journal clusters (knowledge base), while the right side shows the citing journals (application domains), connected by citation paths. Four main citation pathways were identified. Two prominent blue pathways indicate that journals in psychology, education, and health were influenced by work in psychology, education, social sciences and molecular biology and genetics. Notably, citation sources from the psychology, education, and social domain supported research published in both molecular biology and immunology and medicine, medical, clinical domains. These patterns suggest that EEG research in depression spans multiple dimensions, including psychosocial, molecular, and clinical disciplines.

### 3.5 Analysis of co-cited references

**Figure 6:**
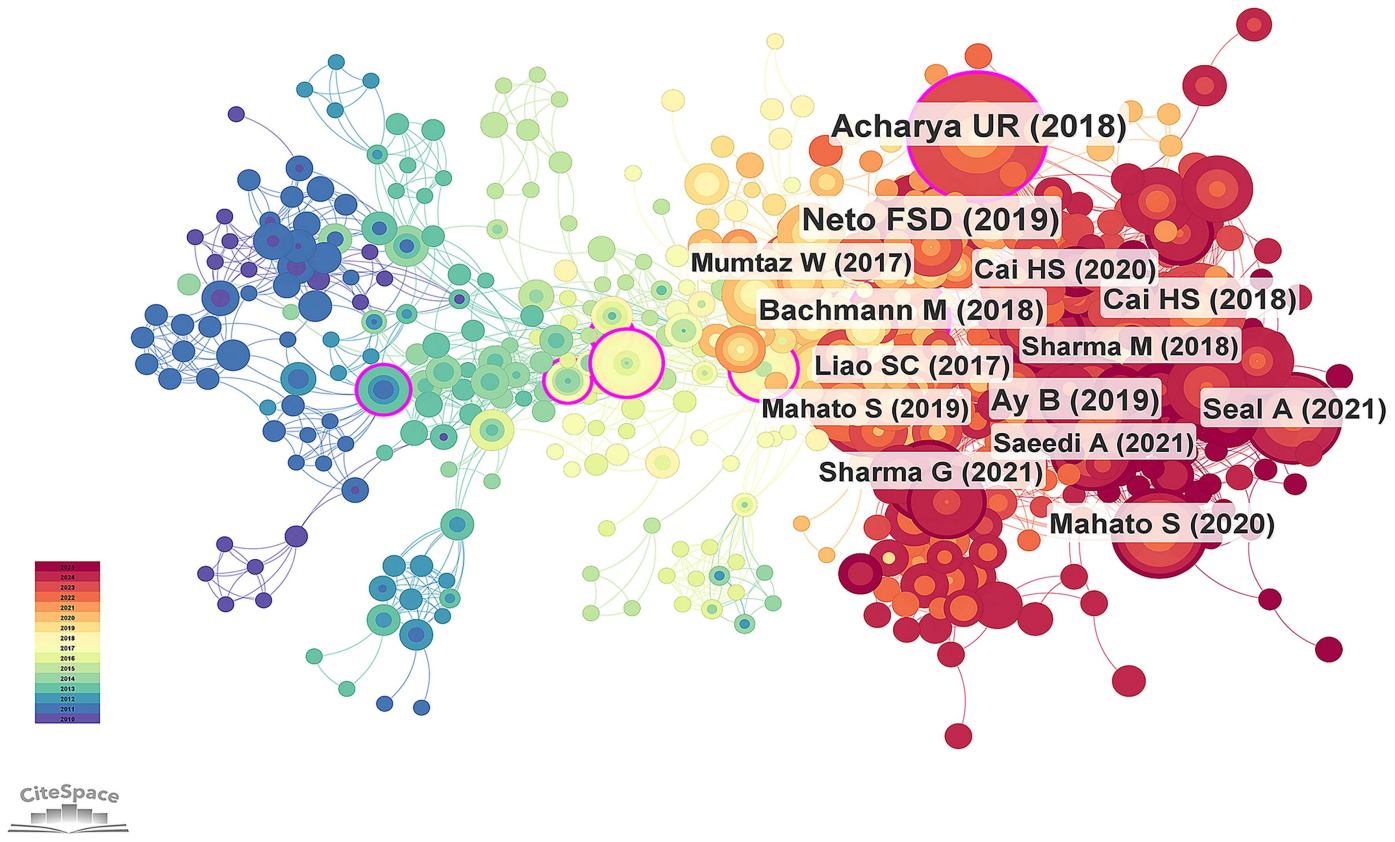
Network visualization diagram for reference co-occurrence.

The top 10 most frequently co-cited references are presented in Table 4, with clinical studies representing the predominant category among them. The article that received the highest frequency of co-citation was published in the *Journal of Neuroscience Methods* in 2004. As shown in Figure 6, the size of each node represents the frequency of co-citation; larger circles indicate a higher number of co-citations. Among them, the study by Acharya et al. (2018) is the most co-cited reference in the field (Acharya et al., 2018). In recent years, the works by Newson and Thiagarajan (2019) and Yasin et al. (2021) have increasingly become focal points of research attention, indicating their emerging influence in this area. Supplementary Figure S4 displays a timeline view of co-cited references from 2005 to 2025. Each curve between two nodes indicates that they were co-cited in the same article. The color of the node border represents the time distribution of citations across different periods. Based on visual analysis with CiteSpace, the timeline view clearly demonstrates the temporal evolution and span of each research cluster. Major clusters were labeled with terms such as “feature extraction,” “QEEG,” “recurrent depression,” “transcranial magnetic stimulation,” “TMS-EEG,” “major depression,” “mild depression,” “EEG microstates,” and “perinatal depression.” Notably, clusters related to TMS-EEG and feature extraction have shown a marked increase in recent years, coupled with a notable rise in citations, highlighting their status as emerging research frontiers.

**Table 4:** The top 10 co-cited references in the field of EEG research related to depression.

Rank	Co-cited references	Citations	Journal	Types
1	EEGLAB: an open source toolbox for analysis of single-trial EEG dynamics including independent component analysis Delorme A, 2004, J. Neurosci. Methods, V134, P9, DOI: 10.1016/J.JNEUMETH.2003.10.009	103	Journal of Neuroscience Methods	Article
2	Classifying depression patients and normal subjects using machine learning techniques and nonlinear features from EEG signal Hosseinifard B, 2013, Comput. Methods Programs Biomed., V109, P339, DOI: 10.1016/J.CMPB.2012.10.008	92	Computer Methods and Programs In Biomedicine	Article
3	Automated EEG-based screening of depression using deep convolutional neural network Acharya UR, 2018, Comput. Methods Programs Biomed, V161, P103, DOI: 10.1016/J.CMPB.2018.04.012	77	Computer Methods and Programs in Biomedicine	Article
4	EEG power, frequency, asymmetry and coherence in male depression Knott V, 2001, Psychiatry Res., V106, P123, DOI: 10.1016/S0925-4927(00)00080-9	76	Psychiatry Research-Neuroimaging	Article
5	Left frontal hypoactivation in depression Henriques JB, 1991, J. Abnorm. Psychol., V100, P535, DOI: 10.1037/0021-843X.100.4.535	72	Journal of Abnormal Psychology	Article
6	A rating scale for depression Hamilton M, 1960, J. Neurol. Neurosurg. Psychiatry, V23, P56, DOI: 10.1136/JNNP.23.1.56	70	Journal of Neurology Neurosurgery and Psychiatry	Article
7	Depression biomarkers using non-invasive EEG: a review Neto FSD, 2019, Neurosci. Biobehav. Rev., V105, P83, DOI: 10.1016/J.NEUBIOREV.2019.07.021	67	Neuroscience and Biobehavioral Reviews	Review
8	EEG biomarkers in major depressive disorder: discriminative power and prediction of treatment response Olbrich S, 2013, Int. Rev. Psychiatry, V25, P604, DOI: 10.3109/09540261.2013.816269	67	International Review of Psychiatry	Article
9	A novel depression diagnosis index using nonlinear features in EEG signals Acharya UR, 2015, Eur. Neurol., V74, P79, DOI: 10.1159/000438457	56	European Neurology	Article
10	A pervasive approach to EEG-based depression detection Cai HS, 2018, Complexity, V0, P0, DOI: 10.1155/2018/5238028	55	Complexity	Article

### 3.6 Keyword co-occurrence and research focus

**Figure 7:**
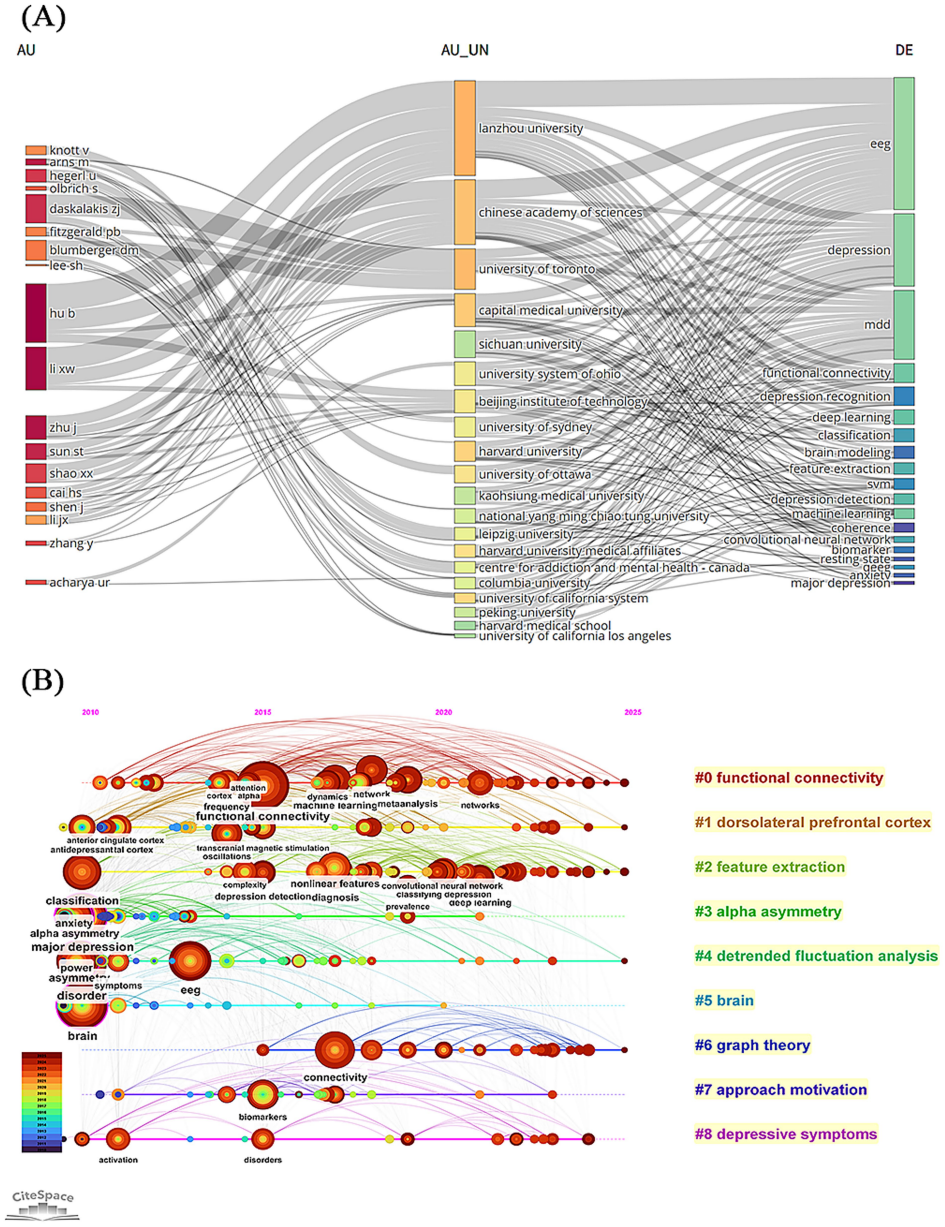
**(A)** The three-field plot on EEG for depression. **(B)** The timeline view of the co-occurrence references network.

**Figure 8:**
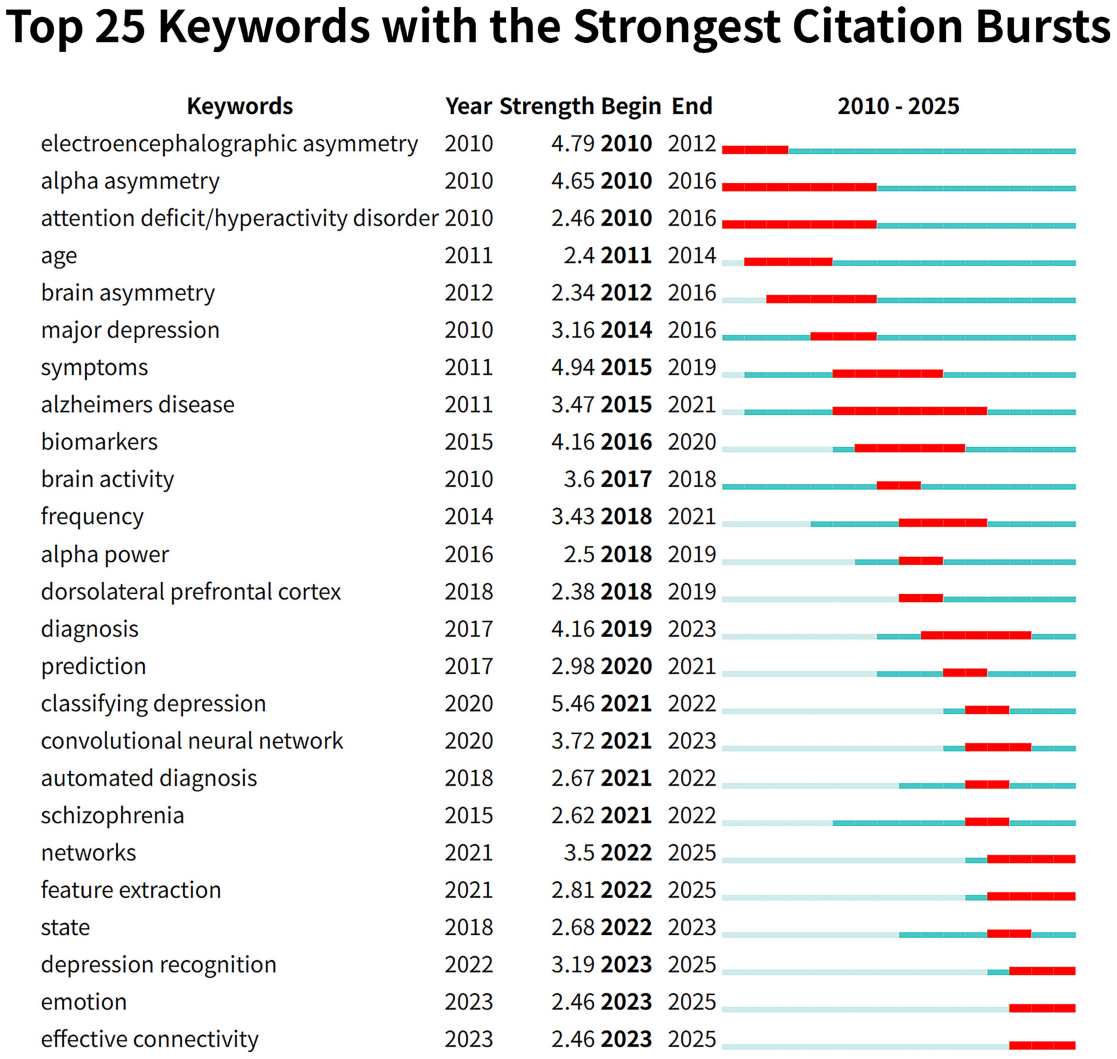
The top 25 keywords ranked by citation burst intensity.

Supplementary Figures S5, S6 show the visual mapping of keyword co-occurrence and clustering. Table 5 presents the most frequently co-occurring keywords. According to co-occurrence frequency and centrality measures, prominent research keywords include major depression, alpha asymmetry, brain, asymmetry, and anxiety. Using the log-likelihood ratio (LLR) algorithm, keywords were grouped into nine major clusters: depressive symptoms, feature extraction, detrended fluctuation analysis, functional connectivity, graph theory, brain, dorsolateral prefrontal cortex, alpha asymmetry, and approach motivation. Generally, a modularity (*Q*) value >0.3 and an average silhouette (*S*) value >0.5 indicate good structural validity of the clustering. In this study, the *Q* value was 0.3741 and the *S* value was 0.7059, indicating high internal consistency and reliability of the clustering results. The three-field plot depicting relationships among authors, institutions, and keywords is shown in Figure 7A. The gray curves represent linkage relationships, with curve thickness corresponding to the frequency of keyword occurrences. The results indicate that researchers from Lanzhou University and the *Chinese Academy of Sciences* have primarily focused on the application of EEG in depression research. Figures 7B, 8 further illustrate the temporal evolution of keyword usage. Early studies primarily concentrated on topics such as alpha asymmetry, detrended fluctuation analysis, and brain. In contrast, recent research has increasingly focused on functional connectivity, dorsolateral prefrontal cortex, feature extraction, and graph theory. Current hotspot keywords include networks, feature extraction, depression recognition, emotion, and effective connectivity.

**Table 5:** Top 10 co-occurring keywords.

Rank	Keywords	Count	Centrality
1	major depression	84	0.2
2	alpha asymmetry	51	0.16
3	brain	83	0.15
4	asymmetry	64	0.13
5	anxiety	48	0.12
6	functional connectivity	85	0.1
7	classification	58	0.08
8	antidepressant	21	0.08
9	disorder	75	0.07
10	frequency	30	0.06

## 4 Discussion

### 4.1 General information related to publications

The studies incorporated in this bibliometric analysis adhere to the minimum criteria established in the preliminary guidelines for conducting bibliometric research (Montazeri et al., 2023). We used the WoSCC database to search for relevant literature published from April 16, 2005 to April 16, 2025, totaling 988 publications, and ultimately included 590. Overall, the annual number of publications in this field has shown a significant upward trend, reflecting growing academic interest and continuous exploration of the value of EEG in depression research. Given the field’s growing research momentum and clinical potential, the number of related publications is expected to continue increasing.

Among the top 10 most productive countries, China and the U. S. hold leading positions, possibly due to the high prevalence of depression in both countries (Heo et al., 2008; Qin et al., 2018). Notably, five of the top 10 most productive institutions are located in China, underscoring the country’s significant influence in this research domain. While the United States does not rank first in publication volume, it leads in citation frequency, highlighting its substantial scientific impact and dominant role in international academic communication and knowledge dissemination. Institutions such as Harvard University and the University of Helsinki have published landmark studies that have laid a strong theoretical foundation for subsequent global research and technological advancements.

At the author level, Hu B. is one of the most prolific scholars in the field. Affiliated with both Beijing Institute of Technology and Lanzhou University, his research focuses on emotional and cognitive computing using multimodal physiological and psychological signals, as well as biosensing technologies. His most influential work was published in *Information Fusion* in 2020, where he developed a depression recognition model using multimodal EEG data under neutral, negative, and positive audio stimuli. The study found that the KNN classifier achieved the highest classification accuracy of 86.98% when combining EEG responses to both positive and negative stimuli (Cai et al., 2020). The top 10 most productive journals accounted for 32.07% of all publications, reflecting the concentration of influence in a few specialized outlets. The *Journal of Affective Disorders* ranked first in the number of publications and also held the highest IF among them. Although *Biological Psychiatry* did not possess the highest IF among the co-cited journals, its citation frequency was markedly superior to that of the other publications. Among all co-cited journals, the *American Journal of Psychiatry* exhibited the highest IF and is esteemed for disseminating high-quality articles that concentrate on the diagnosis and treatment of mental health disorders. Most of the journals listed in Table 3 are primarily oriented toward publishing original clinical research, which is consistent with the dual-map overlay visualization results in Figure 5. Earlier research in this field focused on molecular and genetic mechanisms, but with the development of signal and image processing technologies such as machine learning, research has increasingly shifted toward healthcare and clinical applications (Ahire, 2025; Luo et al., 2025; Xu et al., 2025).

### 4.2 Research foundation

Citation frequency reflects the attention and academic recognition an article receives (Brandt et al., 2019). The 10 most frequently co-cited references predominantly originate from reputable academic journals (Table 4). Among them, the top co-cited reference was an article that proposed EEGLAB, an open-source toolbox running in the Matlab environment, aiming to provide a comprehensive and user-friendly solution for EEG data analysis and promoting the transformation of EEG research from traditional analysis methods to more advanced techniques (Delorme and Makeig, 2004). The second most co-cited reference was published by Hosseinifard et al. (2013) in *Computer Methods and Programs in Biomedicine* (Hosseinifard et al., 2013). The authors analyzed EEG signals from 45 patients with depression and 45 healthy controls, demonstrating the effectiveness of nonlinear features in distinguishing between the two groups. Significant differences in the alpha band were observed, and correlation dimension emerged as a powerful feature for EEG signal analysis. Notably, the study used independent testing and showed high classification accuracy, with logistic regression outperforming other classifiers. The third most frequently co-cited reference was the study by Acharya et al. (2018), which proposed an automated depression screening model based on convolutional neural networks (CNNs). This model eliminates the need for manual feature extraction and has potential for computer-aided diagnosis of depression. The study also found that EEG signals from the right hemisphere were more specific for diagnosing depression (Acharya et al., 2018). These foundational studies have provided both theoretical and empirical support for the development of EEG-based depression research.

### 4.3 Research hotspots

Co-occurrence and burst keyword analysis using CiteSpace and VOSviewer revealed a clear shift in research focus, from basic electrophysiological mechanisms to clinical applications. In the early stages, research primarily focused on electroencephalographic asymmetry and alpha asymmetry, which contributed to understanding the pathophysiological basis of depression. Over time, keywords such as “diagnosis,” “prediction,” and “classifying depression” gained prominence, indicating a transition toward practical clinical use. Among the 53 clinical studies retrieved from PubMed, 38 (71.7%) focused on the clinical application value of EEG, covering diagnostic markers (such as (Deng et al., 2015), where the global efficiency and small-worldness of brain networks in depressed patients were found to be reduced, and the topological structure disorder could serve as a diagnostic feature), efficacy prediction [such as (Oakley et al., 2023), β-band phase synchronization index predicting sertraline response], and treatment monitoring (such as George et al., 2023), EEG synchronization TMS for treatment-resistant depression). More recently, keywords like “networks,” “feature extraction,” “depression recognition,” “emotion,” and “effective connectivity” have become research focal points, particularly in the context of extracting EEG features related to cognitive dimensions of major depressive disorder (MDD).

Keyword clustering revealed several major themes including “depressive symptoms,” “feature extraction,” “detrended fluctuation analysis,” “functional connectivity,” “graph theory,” “brain,” “dorsolateral prefrontal cortex,” “alpha asymmetry,” and “approach motivation.” From 2005 to 2025 a central research aim has been to identify depression-related EEG patterns and extract features that could serve as objective biological markers for diagnosis and assessment. Li et al. (2025) recruited 97 drug-naïve first-episode MDD patients and recorded 10-min resting-state EEG data with eyes closed. Using 32-channel EEG caps and a sampling rate of 1,000 Hz the study applied the FOOOF algorithm to separate periodic and aperiodic components and extract personalized spectral features. Significant differences were found in alpha and beta band power alpha asymmetry and the aperiodic offset between patients and controls. The severity of depression was correlated with the aperiodic offset. Liu et al. (2022) recorded EEG data in eyes-open and eyes-closed conditions from 30 first-episode MDD patients and found widespread increases in beta and gamma bands in the MDD group suggesting that these frequency bands could serve as potential biomarkers for early-stage depression.

With advances in computational and analytical techniques, EEG research has moved beyond basic signal descriptions toward more complex network-level analysis. Keywords such as “detrended fluctuation analysis,” “functional connectivity,” and “graph theory” reflect this methodological evolution. Detrended fluctuation analysis captures nonlinear signal properties, functional connectivity reveals interactions between brain regions, and graph theory describes the topological organization of brain networks. These methods have facilitated investigation into specific brain regions, such as the dorsolateral prefrontal cortex (DLPFC), which is involved in emotion regulation and cognitive control (Segal and Elkana, 2023) Using TMS-EEG, Li et al. (2023) investigated cortical excitability of the DLPFC in 41 MDD patients and 42 healthy controls. Stimulation was applied to the F3 electrode site (representing the left DLPFC) using a Magstim TMS device and a 64-channel EEG system. The study found reduced prefrontal excitability in MDD patients, which correlated with depression severity (Li et al., 2023). The study of alpha asymmetry is also crucial within this field. Patients with depression often exhibit reduced frontal alpha power with asymmetrical distribution, decreased activity in the left prefrontal cortex and increased activity in the right, suggesting a potential neural marker for depression that may assist clinicians in more objective diagnosis (Zhao et al., 2025).

EEG-based studies on depression have achieved substantial progress. For instance, researchers have consistently observed reduced alpha and increased theta power in patients. As a non-invasive, real-time brain monitoring technique, EEG provides multidimensional insights that facilitate early screening, symptom monitoring, and clinical assessment. In the following sections, we elaborate on the role of EEG in depression diagnosis from five perspectives: EEG frequency bands, functional connectivity, EEG topography, machine learning algorithms, and ERPs.

### 4.4 The role of EEG in the diagnosis and classification of depression

#### 4.4.1 Based on the characteristics of EEG frequency bands

EEG provides frequency-specific insights into the brain’s functional state and has been increasingly applied to investigate the pathophysiological mechanisms and assist in the diagnosis of depression. Different EEG frequency bands reflect distinct neuronal dynamics and are associated with various functional connectivity patterns across brain regions. For example, alpha waves (8–12 Hz) are most prominent in the occipital region during eyes-closed resting states and are typically considered an electrophysiological marker of cortical “idling” or inhibition (Tang et al., 2012). In patients with depression, frontal alpha activity often exhibits pronounced left–right asymmetry, characterized by increased power in the left prefrontal cortex and decreased power in the right (Saletu et al., 2010). This pattern, known as frontal alpha asymmetry (FAA), has been associated with negative affective bias and motivational withdrawal, and is considered a potential neurophysiological marker of depression (Thibodeau et al., 2006).

However, the clinical generalizability of FAA remains controversial due to its variability across individuals and susceptibility to confounding factors such as sex. While some studies have reported gender-related differences in FAA (Jesulola et al., 2017), others have failed to replicate these findings (Smith et al., 2018). Additionally, FAA patterns may overlap with anxiety symptoms, making it difficult to distinguish between depression and anxiety in comorbid patients (Nusslock et al., 2018). Nevertheless, FAA has shown potential in differentiating unipolar from bipolar depression, identifying anxiety disorders, and recognizing occupational burnout, especially when integrated with multimodal neuroimaging techniques (van der Vinne et al., 2017). Before FAA can be adopted as a standardized clinical biomarker, however, further validation through large-sample, multi-center studies with standardized acquisition protocols is necessary.

Theta waves (4–8 Hz) represent another key frequency band and are particularly active along the hippocampal-prefrontal circuitry. Theta activity is prominently involved in tasks related to memory processing and emotional regulation (Zangbar et al., 2020). Mitchell et al. (2008) reported that frontal midline theta (FM-theta) is strongly correlated with task difficulty, cognitive load, and performance during working memory, spatial navigation, and episodic memory tasks. Coherence in theta oscillations between different brain regions, particularly between the frontal cortex and hippocampus, suggests a critical role for theta waves in inter-regional information integration. Combining spectral features of alpha and theta bands has demonstrated promising results in distinguishing individuals with depression from healthy controls (Chang and Choi, 2023). When coupled with machine learning algorithms, diagnostic performance has been further enhanced (Chiang et al., 2023; Zhang B. T. et al., 2023; Zhang X. et al., 2023), paving the way for more objective and automated diagnostic approaches.

Compared to alpha and theta bands, beta (13–30 Hz) and delta (1–4 Hz) frequencies have received less attention in depression research, though their diagnostic potential is gaining recognition. In a study involving 32 healthy controls and 33 patients with depression, Li et al. (2017) found significantly elevated phase synchronization index (PSI) in the beta band among the depressed group. This heightened beta-band synchrony may reflect compensatory hyperactivation in neocortical circuits, potentially impacting cognitive flexibility (Li et al., 2017). In another study, Armitage et al. (2001) compared delta wave activity during early non-rapid eye movement (NREM) sleep in eight adolescent females with depression and eight healthy controls. The depressed group exhibited significantly reduced delta amplitude and power, suggesting impaired sleep-related cortical activity, possibly moderated by sex-related factors.

#### 4.4.2 Based on the connectivity of EEG

Functional EEG connectivity has become a vital approach in investigating aberrant brain function in depression. By quantifying the synchronization of neural activity between different brain regions, this method helps uncover disruptions in underlying neural circuits. Common connectivity measures include coherence, phase locking value (PLV), Granger causality, and phase lag index (PLI), each reflecting signal dependencies in the frequency and time domains. Numerous studies have shown that patients with MDD exhibit significantly weakened connectivity between the prefrontal cortex and the limbic system at rest, particularly in the alpha band (8–13 Hz), indicating a marked reduction in coherence between the left prefrontal region and other brain areas (Leuchter et al., 2012). This may be closely related to impaired emotion regulation, anhedonia, and attentional disorders (Knyazev, 2012).

In recent years, the introduction of graph theory analysis methods has made it possible to evaluate the topological structure of EEG networks. Studies have found that the brain networks in patients with MDD exhibit characteristics such as reduced small-worldness, decreased global efficiency, and disordered modular structure, indicating impaired neural information transmission efficiency (Kabbara et al., 2022; Teng et al., 2024).

#### 4.4.3 Based on the EEG topography

EEG topographic mapping visualizes brain activity by interpolating the spatial distribution of power or frequency features across electrode sites. In depression, topographic maps reveal abnormal activation patterns in specific brain regions, particularly in the distribution of alpha and theta bands. The most prominent metric is FAA, considered a potential biomarker for depression (Henriques and Davidson, 1991). Depressed individuals often show increased alpha power (indicative of decreased cortical activity) in the left prefrontal cortex, a pattern associated with negative affect and motivational withdrawal (Thibodeau et al., 2006).

Topographic mapping is also widely applied to evaluate post-stimulus EEG responses. For example, Canli et al. (2004) observed that individuals with depression displayed reduced activation in response to positive emotional words and heightened activation to negative words during an affective judgment task. This emotion-related bias exhibited characteristic spatial distributions on the EEG map.

#### 4.4.4 Integrating machine learning algorithms

Machine learning (ML) algorithms have been increasingly utilized in EEG-based depression research, offering automated pipelines for feature extraction, classification, and outcome prediction. Traditional classifiers such as support vector machines (SVM), random forests (RF), and k-nearest neighbors (k-NN), remain widely used for their simplicity and interpretability, particularly in small-sample settings. For instance, Acharya et al. (2018) demonstrated the feasibility of using handcrafted EEG features with SVM to distinguish depressed individuals with high accuracy.

In recent years, deep learning models have gained prominence due to their ability to learn complex spatiotemporal features directly from raw EEG signals. CNNs, LSTM networks, and hybrid CNN-LSTM architectures have shown superior performance across multiple datasets. For example, Zhang B. T. et al. (2023) and Zhang X. et al. (2023) proposed a hybrid CNN-LSTM framework for emotion classification using EEG, achieving over 90% accuracy and demonstrating the model’s ability to capture both spatial and temporal dynamics effectively. In addition, robust validation strategies are essential to ensure generalizability. Common methods include k-fold cross-validation, leave-one-subject-out (LOSO), and nested cross-validation. Craik et al. (2019) reviewed EEG deep learning studies and emphasized that the lack of consistent validation standards leads to inflated performance metrics. Roy et al. (2019) further advocated for LOSO and external validation using independent cohorts, especially in clinical applications.

Model interpretability is another growing priority. Although deep learning models improve accuracy, their “black-box” nature limits clinical acceptability. To address this, explainable AI (XAI) methods have been adopted. Sturm et al. (2016) applied layer-wise relevance propagation (LRP) to highlight which EEG segments contributed to single-trial classification. Mayor Torres et al. (2023) systematically evaluated saliency-based methods such as Grad-CAM and LRP in EEG-based classification tasks, contributing valuable insights for practical deployment in clinical neurotechnology. Several studies have proposed and evaluated XAI methods such as saliency maps, Grad-CAM, and SHAP for interpreting deep learning models applied to EEG-based emotion recognition, thereby promoting clinical integration (Samek et al., 2021).

#### 4.4.5 Based on the event-related potentials

ERPs are time-locked electrophysiological responses to specific stimuli, representing the brain’s dynamic processing of sensory, cognitive, and emotional events, often referred to as the “electrical fingerprints” of cognition. In the context of depression diagnosis, several ERP components have shown diagnostic value. Among these, the P300 component is most widely studied. Occurring approximately 300 ms after stimulus onset, P300 is associated with cognitive decision-making, attentional allocation, and working memory. Studies consistently report prolonged P300 latency and reduced amplitude in depressive patients. For example, Kemp et al. (2010) found an average P300 amplitude reduction of approximately 1.0 μV (equivalent to 15–25% of the healthy control group) and latency increases of 10–30 ms, reflecting impaired cognitive speed and information processing efficiency.

The N400 component, primarily involved in semantic processing, is another ERP marker of interest. Its amplitude is modulated by the congruency and complexity of semantic stimuli (Lau et al., 2008). Abnormal N400 responses are frequently observed in depression (Griffin and Schnyer, 2020; Xu et al., 2023). Kiang et al. (2017) found that MDD patients exhibited significantly reduced N400 amplitudes when processing negative adjectives, while responses to positive and neutral words were comparable to healthy controls. This suggests that negative semantic content more strongly activates self-referential negative representations in MDD patients, indicating enhanced connectivity within the negative semantic memory network.

### 4.5 Methodological evolution and clinical translation

EEG-based depression research has evolved from early descriptive analysis, such as FAA and ERPs, to advanced approaches including functional connectivity, graph theory, and topographic mapping. While FAA initially offered potential as a biomarker, its clinical reliability remains limited due to inter-individual variability (Thibodeau et al., 2006; Jesulola et al., 2017; van der Vinne et al., 2017). The adoption of connectivity metrics and graph theory has allowed a deeper understanding of large-scale brain network disruptions in depression, such as reduced global efficiency and small-worldness, which are linked to symptom severity and treatment outcomes (Deng et al., 2015; Kabbara et al., 2022). More recently, machine learning such as CNN and LSTM models, has enabled automated EEG-based classification with high accuracy, reducing the need for manual preprocessing and improving scalability (Acharya et al., 2018; Chiang et al., 2023; Ahire, 2025). These advances open the door to real-time, accessible EEG tools for early diagnosis and monitoring in clinical settings.

These methodological innovations are moving EEG toward practical clinical use in precision psychiatry. However, widespread implementation will require standardization, external validation, and improved interpretability to ensure clinical reliability.

## 5 Limitations

As far as we know, this study stands as the first bibliometric analysis specifically targeting EEG-related studies in depression. However, several important limitations should be acknowledged. Although the WoSCC and PubMed are the most widely utilized academic databases, they do not encompass all published literatures. To improve the accuracy of our analysis, we employed a title-based keyword as opposed to a topic-based search. While this approach ensures a certain level of specificity, it inevitably introduces limitations. Moreover, restricting the analysis to English-language publications may result in language bias and restrict the generalizability of our findings. The high quality research recently released may not have received sufficient attention. Although the number of EEG studies on depression has been on the rise in recent years, the total number of articles is still relatively small, highlighting the necessity of continued research.

Despite the methodological advancements summarized in this review, several practical challenges continue to impede the clinical translation of EEG-based tools for depression. First, data heterogeneity remains a critical barrier: EEG recordings vary considerably across acquisition hardware, electrode montages, preprocessing pipelines, and participant conditions (such as eyes-open and eyes-closed), limiting the generalizability of machine learning models across datasets and clinical environments (Cassani et al., 2018). Second, reproducibility issues persist, as many studies lack standardized benchmarks, openly accessible datasets, or transparent reporting of model training pipelines, which complicates cross-study comparisons and replication efforts (Snoek et al., 2018). Third, the absence of standardized protocols, including electrode placement, recording duration, and task paradigms, continues to hinder scalability and real-world integration of EEG tools in psychiatric care (Krigolson et al., 2017). Addressing these limitations will require coordinated efforts to create shared EEG data repositories, establish consensus guidelines, and promote multi-center validation studies to ensure robustness, transparency, and clinical relevance.

## 6 Conclusion

From 2005 to 2025, the application of EEG in depression research has continued to grow, with China and the U. S. being the main research forces. Research hotspots have shifted from traditional indicators (such as alpha wave asymmetry, ERPs) to brain network analysis, topographic mapping, and artificial intelligence technology, reflecting a more precise and individualized exploration of the mechanism of depression. EEG has shown great potential in diagnosis, treatment monitoring, and mechanism research, but still faces problems such as sample heterogeneity and insufficient model validation. In the future, multi-center large-sample studies should be strengthened, the integration of EEG with multimodal neuroimaging should be promoted, data processing procedures should be standardized, and the transparency and reproducibility of models should be improved. At the same time, longitudinal studies should be conducted to evaluate the application value of EEG biomarkers in predicting treatment efficacy. Strengthening interdisciplinary cooperation and data sharing will help the clinical transformation of EEG in depression research.

## Data Availability

The original contributions presented in the study are included in the article/Supplementary material, further inquiries can be directed to the corresponding authors.

## Footnotes

^1^ www.biorender.com

^2^ https://donghaoren.org/charticulator/

^3^ https://www.vosviewer.com/getting-started

^4^ https://citespace.podia.com/

^5^ https://data.worldbank.org/

## Glossary

MDD: Major depressive disorder
EEG: Electroencephalogram
DALYs: Disability-adjusted life years
YLDs: Years lived with disability
WHO: World Health Organization
ERPs: Event-related potentials
fMRI: Functional magnetic resonance imaging
WoSCC: Web of Science Core Collection
IF: Impact factor
JCR: Journal Citation Reports
GDP: Gross domestic product
LLR: Log-likelihood ratio
DLPFC: Dorsolateral prefrontal cortex
FAA: Frontal alpha asymmetry
FM-theta: Frontal midline theta
NREM: Non-rapid eye movement
PSI: Phase synchronization index
PLV: Phase locking value
PLI: Phase lag index
ML: Machine learning
CNNs: Convolutional neural networks
LSTM: Long short-term memory
SVM: Support vector machines
RF: Random forests
k-NN: k-nearest neighbors
LOSO: Leave-one-subject-out
XAI: Explainable AI
LRP: Layer-wise relevance propagation
