# Supplementary material for: The application of electroencephalogram in depression research: bibliometric and technological application analysis from 2005 to 2025
Source: Front Neurosci. 2025 Aug 21;19:1653693. doi: 10.3389/fnins.2025.1653693 (PMC12408689; doi:10.3389/fnins.2025.1653693)
Supplement: Supplementary file 1 [file Data_Sheet_1.docx]

**Appendix 1**

**Figure S1**. Analysis of countries/regions in the field of EEG for depression.


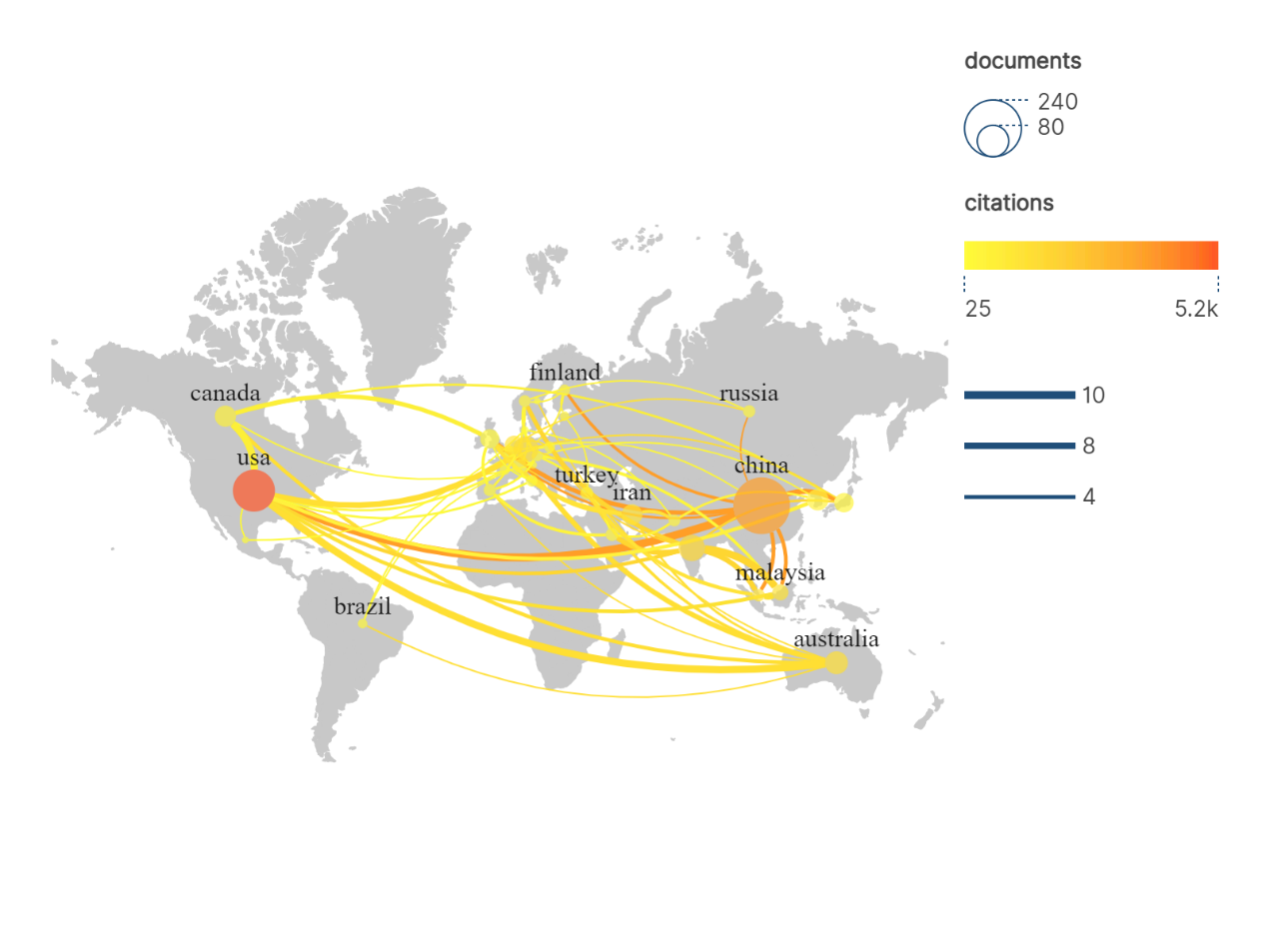


**Figure S1.** Publications and citations in the top ten most productive countries/regions and institutions.

| Rank | country | publications | citations | average citations | institutions | publications | citations | average citations |
| --- | --- | --- | --- | --- | --- | --- | --- | --- |
| 1 | China | 214 | 3761 | 17.57 | lanzhou university | 45 | 1509 | 33.53 |
| 2 | USA | 118 | 5201 | 44.08 | Chinese Academy of Sciences | 30 | 983 | 32.77 |
| 3 | India | 46 | 1728 | 37.57 | Beijing Institute of Technology | 20 | 260 | 13.00 |
| 4 | Germany | 42 | 1310 | 31.19 | Capital Medical University | 19 | 762 | 40.11 |
| 5 | Australia | 35 | 1339 | 38.26 | University of Toronto | 16 | 400 | 25.00 |
| 6 | South Korea | 31 | 453 | 14.61 | Leipzig University | 10 | 325 | 32.50 |
| 7 | Canada | 30 | 691 | 23.03 | University of Tehran | 10 | 533 | 53.30 |
| 8 | Iran | 29 | 1141 | 39.35 | Chinese Institute for Brain Research | 9 | 558 | 62.00 |
| 9 | Japan | 25 | 440 | 17.60 | Utrecht University | 9 | 558 | 62.00 |
| 10 | United Kingdom | 24 | 277 | 11.54 | Ngee Ann Polytechnic | 9 | 1054 | 117.11 |

**Figure S2**. Overlay visualization map of productive institutions co-authorship analysis.


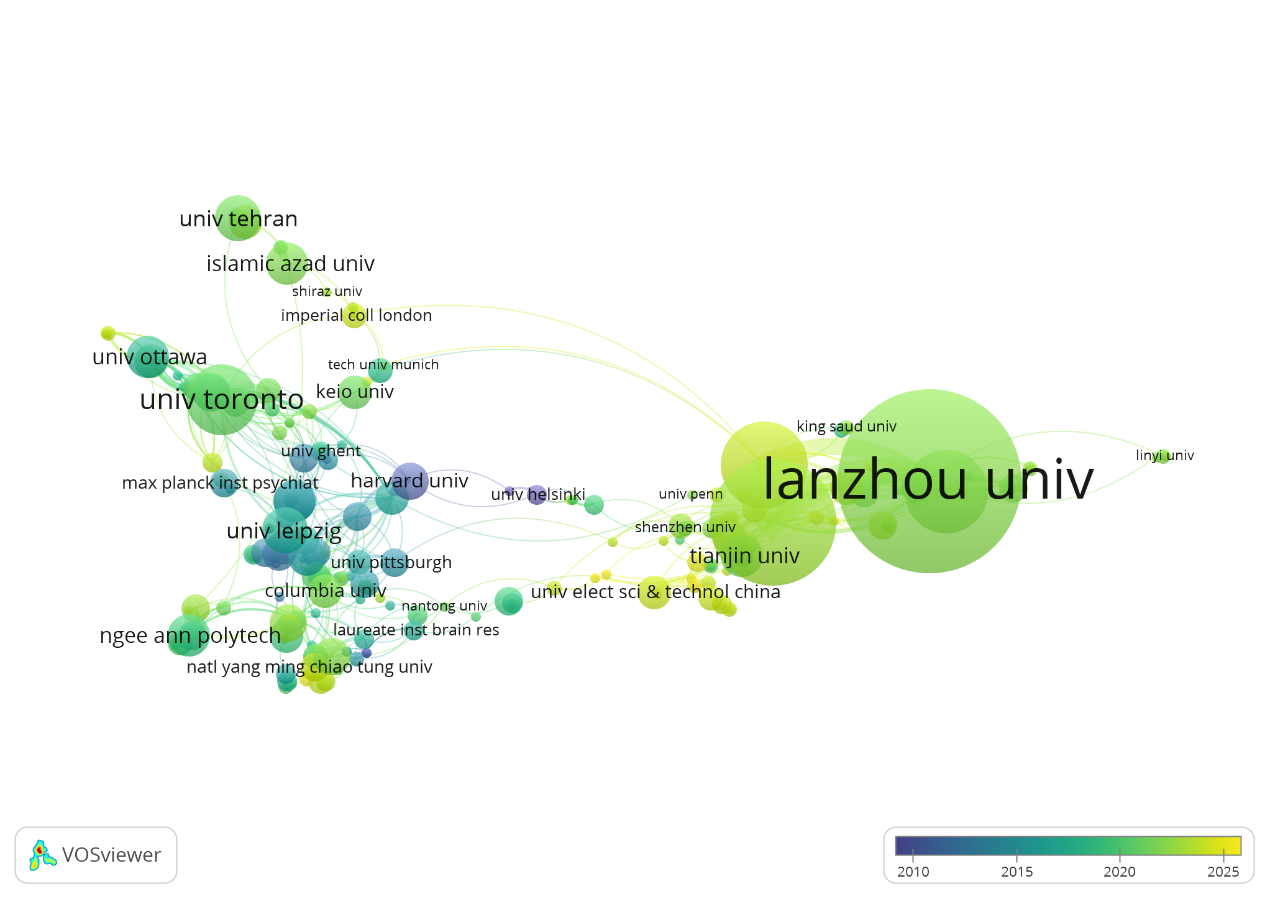


**Appendix 2**

Figure S3. Core journals classified according to Bradford's Law.


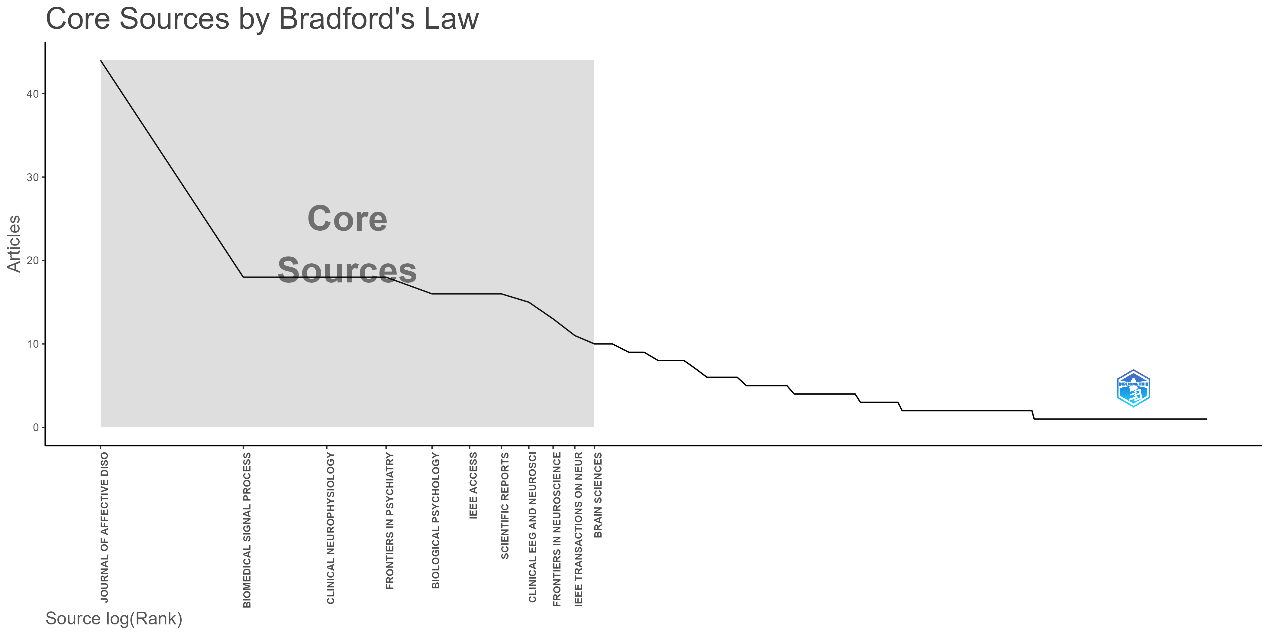


**Appendix 3**

Figure S4. The timeline view of the co-cited references network.

**
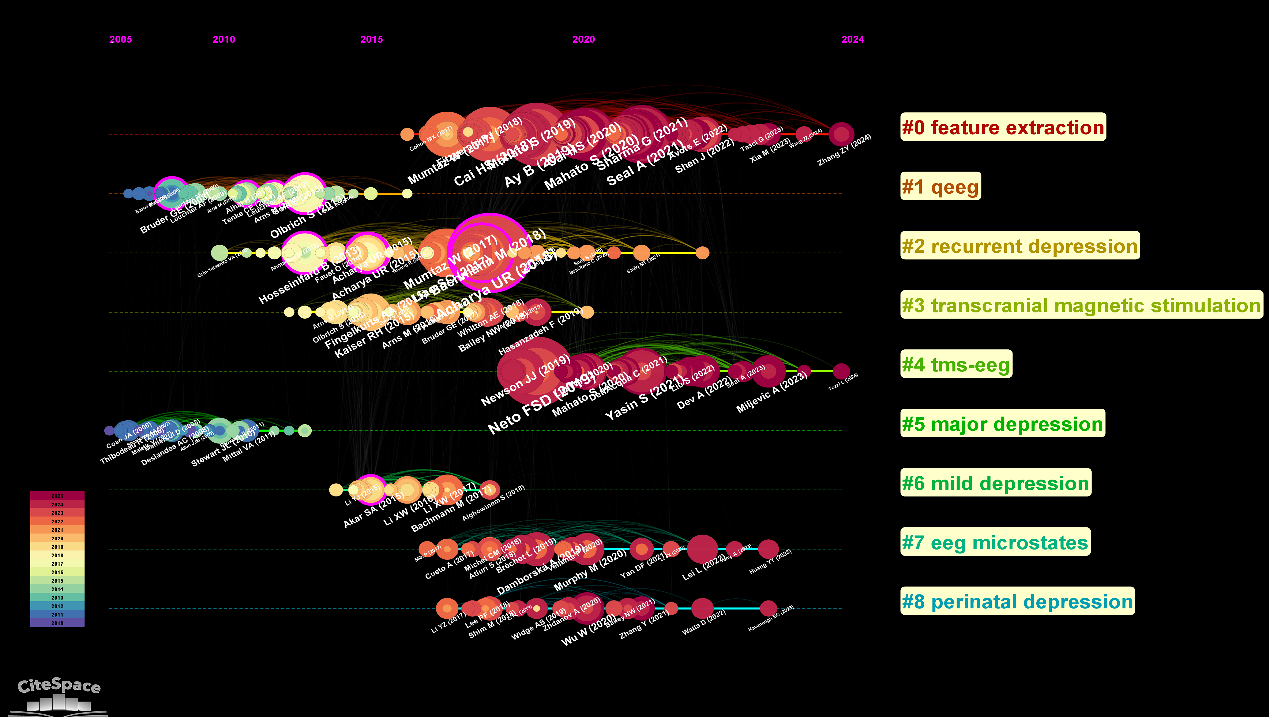
**

**Appendix 4**

Figure S5. Network visualisation diagram for keyword co-occurrence.

**
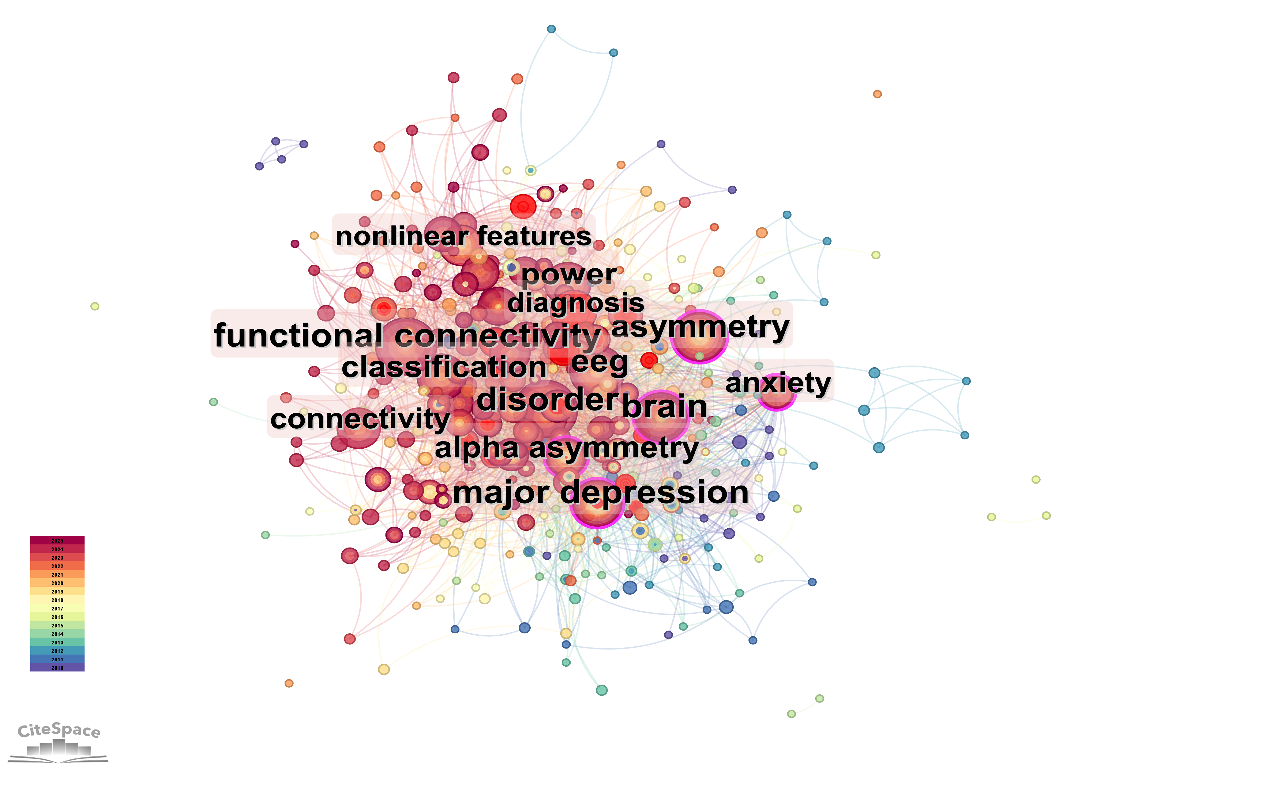
**

Figure S6. Network visualisation diagram for keyword clustering.

**
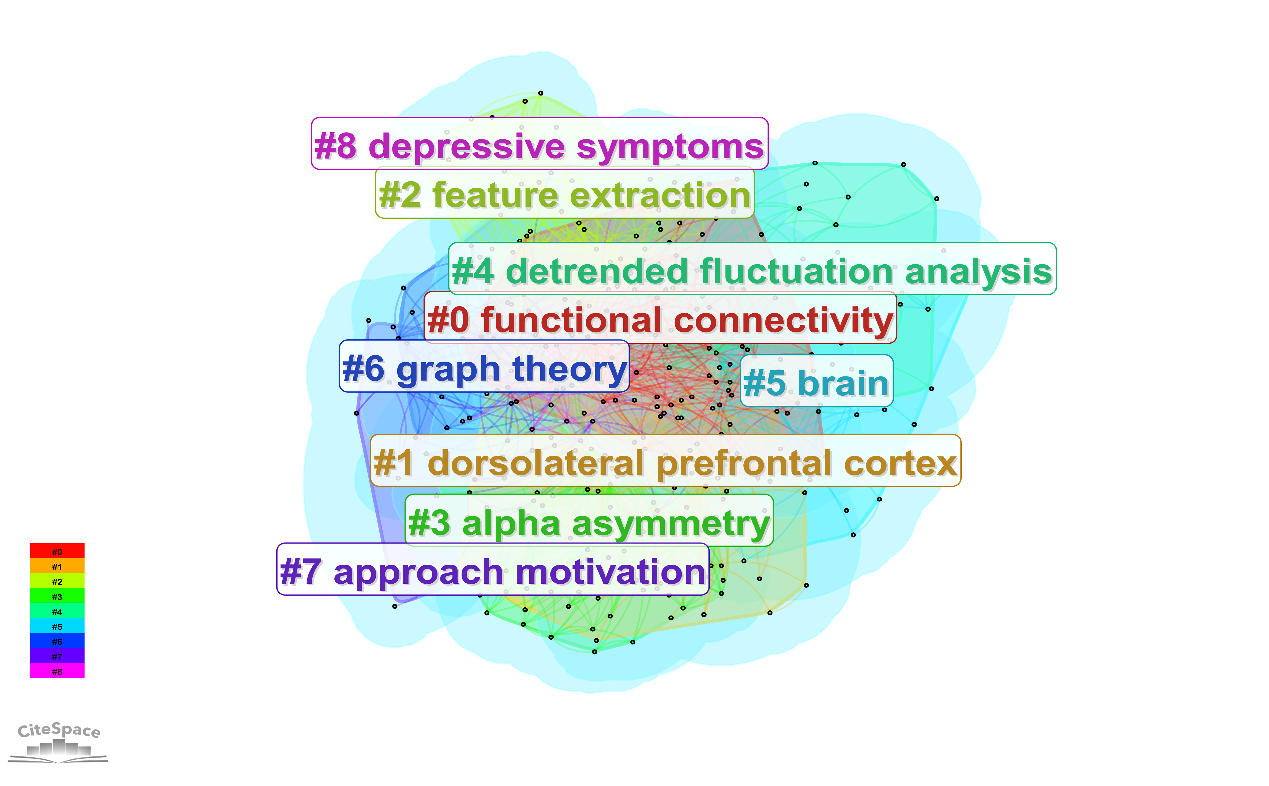
**
